# Neuronal Senescence in the Aged Brain

**DOI:** 10.14336/AD.2023.0214

**Published:** 2023-10-01

**Authors:** Shu-Min Chou, Yu-Hsin Yen, Fang Yuan, Su-Chun Zhang, Cheong-Meng Chong

**Affiliations:** ^1^Program in Neuroscience & Behavioral Disorders, Duke-NUS Medical School, 169857 Singapore, Singapore.; ^2^Department of Neuroscience, Department of Neurology, Waisman Center, University of Wisconsin-Madison, Madison, WI 53705, USA.; ^3^State Key Laboratory of Quality Research in Chinese Medicine, Institute of Chinese Medical Sciences, University of Macau, Macao, China.

**Keywords:** aging, neuronal senescence, proteostasis, redox balance, calcium dynamics

## Abstract

Cellular senescence is a highly complicated cellular state that occurs throughout the lifespan of an organism. It has been well-defined in mitotic cells by various senescent features. Neurons are long-lived post-mitotic cells with special structures and functions. With age, neurons display morphological and functional changes, accompanying alterations in proteostasis, redox balance, and Ca^2+^ dynamics; however, it is ambiguous whether these neuronal changes belong to the features of neuronal senescence. In this review, we strive to identify and classify changes that are relatively specific to neurons in the aging brain and define them as features of neuronal senescence through comparisons with common senescent features. We also associate them with the functional decline of multiple cellular homeostasis systems, proposing the possibility that these systems are the main drivers of neuronal senescence. We hope this summary will serve as a steppingstone for further inputs on a comprehensive but relatively specific list of phenotypes for neuronal senescence and in particular their underlying molecular events during aging. This will in turn shine light on the association between neuronal senescence and neurodegeneration and lead to the development of strategies to perturb the processes.

## 1. Introduction

Aging remains an inevitable part of human life. Aging is a process with progressive decline in the intrinsic physiological states or functions. Cellular senescence is a highly complicated terminal state that occurs throughout the lifespan starting from the embryonic stage, where it contributes to tissue development, to adulthood, where it prevents propagation of damaged tissues or tumors in addition to its contribution to aging [1]. The concept of cellular senescence was first proposed to describe the limited replicative ability in human fibroblasts [2]. With further exploration, it was found that senescence is not restricted to mitotic cells but also occurs in post-mitotic cells like neurons [3].

Neurons are long-lived with human neurons surviving for several decades. With aging, cellular damage products, misfolded proteins, and toxins accumulate and result in alterations in the functional properties of neurons such as a decline in their function and increased vulnerability to disease and pathology [3, 4]. An obvious sign seen in the elderly is the decline in motor, sensory, memory, and cognitive functions [5, 6]. However, it is ambiguous whether these neuronal changes belong to the features of neuronal senescence.

Much of what we have learnt about cellular senescence is established through the studies of mitotic cells. There are a host of senescent features proposed for mitotic cells, including telomere attrition, DNA damage, loss of lamin B1, increased level of senescence-associated β-galactosidase (SA-β-gal), dysfunctional mitochondria with increased production of reactive oxygen species (ROS), accumulation of macromolecule aggregates, and the senescence-associated secretory phenotypes (SASP) [1]. Currently, scientists borrow these same “senescence phenotypes” to define neuronal senescence. Although most of these features are also seen in neurons (Table 1), the underlying mechanisms of their existence may be different. For example, neurons from old animals exhibit an increase of typical biomarkers for cellular senescence in mitotic cells such as cell-cycle inhibitors p16 and p21, but in neurons, these findings are associated with damage accumulation [3, 7, 8]. These findings hint that senescence-like phenotypes may result from a cellular stress response. In addition, while generally applicable across mitotic and post-mitotic cells, significant differences do exist given the unique cellular and functional features of neurons. The declines in neuronal functions are linked to age-related changes in neuronal structure and synaptic transmission. Highly associated with changes in neuronal structures is the shrinkage in brain volume in the aged brain [9] but with no obvious loss of overall neuronal number unlike in neurodegenerative diseases such as Parkinson’s disease (PD), Huntington's disease (HD), and Alzheimer’s disease (AD) [10], highlighting the difference in neuronal changes between aging and neurodegeneration. Thus, the identification of aging-induced neuronal changes and the potential underlying molecular processes will help us to define neuronal senescence. It will be instrumental to our understanding of the aging process in the nervous system and its relation to neurodegeneration. It may also guide us to develop approaches to decelerate the so far inevitable aging process to delay or slow the development of neurodegenerative disorders.

**Table 1:** Comparison of common senescent features between mitotic cells and neurons.

Senescent features	Mitotic cells	Neurons	Species & References
DNA damage	√	√	human (12, 13, 20), mouse (3, 15, 17), rat (16, 19)
Dysfunctional mitochondria	√	√	human (20), mouse (24), rat (23)
SASP	√	√	mouse (3, 25), rat (16)
Nuclear morphology changes (loss of lamin-B1)	√	√	mouse (35), rat (16, 36)
Macromolecule aggregates (Accumulated lipofuscin)	√	√	human (40), rat (16)
Cell-cycle inhibitors	√	√	mouse (8), rat (16)
SA-β-gal	√	√	mouse (3, 25), rat (16, 36)

In this review, we attempt to identify and categorize the cellular and functional changes relatively specific to neurons in the aged brain and further define them as features of neuronal senescence through comparison with common senescent features. Given the limited studies on neuronal senescence, the features we summarized here are largely from analysis in the aging nervous system, most of which are associated with stress. We also summarize the aging-induced functional decline of intracellular homeostatic systems with a focus on proteostasis, redox balance, and Ca^2+^ homeostasis in neurons to explain their role as main drivers of neuronal senescence.

## 2. Senescent changes common to mitotic cells and neurons

Although neurons do not have certain features of replicative senescence such as telomere attrition, aged neurons share common senescent features such as DNA damage, dysfunctional mitochondria, SASP, nuclear morphological changes, accumulation of macromolecule aggregates, increased level of SA-β-gal, and cell-cycle inhibitors (Table 1) [3].

DNA damage is a well-known mediator for cellular senescence in mitotic cells mainly due to replication missteps and various stressors such as oncogenic stress and oxidative stress [11]. Markers of DNA damage are also accumulated in aged neurons. Sequencing of single neurons from the hippocampus and prefrontal cortex of healthy individuals aged 4 months to 82 years-old revealed that somatic single-nucleotide variant increased approximately linearly with age [12]. A recent study used the more precise method named nanorate sequencing and found that neurons accumulated DNA mutations at a constant rate throughout human life [13]. DNA double strand breaks (DSBs) are the most severe type of DNA damage, mainly connected with cellular senescence [14]. γH2AX is widely used as a marker of DSBs, which is observed in Purkinje cells and cortical neuron from old mice [3, 15, 16]. Other DNA damage such as DNA base modifications and single strand breaks (SSBs) mainly result from oxidative stress. In aged mouse brains, hippocampal pyramidal neurons, granule neurons, and cerebellar granule neurons exhibit an increase in the levels of nuclear DNA SSBs [17] and such SSBs often accumulate at genomic enhancers in neurons [3, 18]. In addition, 8-oxoguanine (8-oxoG) lesions are accumulated in the genomes of aged rat neurons [19]. DNA damage is also observed in mitochondria in neurons from aged individuals [20]. Given the post-mitotic nature and the high demand on oxidative phosphorylation in neurons, DNA damages seen in neurons are likely due to the exposure to high levels of free radicals [21].

Accumulation of dysfunctional mitochondria commonly occurs in senescent cells [22]. This is particularly obvious for neurons which rely on their abundant mitochondria to satisfy their high-energy demand. Ultrastructurally, mitochondria in aged rat neurons have dominant morphological changes such as broken cristae, vacuolization, and accumulation of paracrystalline inclusions [23]. In old mice, optic nerve axons have longer and thicker mitochondria as well as reduced ATP production, accompanied by increased levels of lipid peroxidation, nitric oxide, and protein nitration [24]. In addition, high levels of mitochondrial DNA deletions are observed in neurons from aged individuals [20], revealing that mitochondria dysfunction may result from mitochondrial damage occurs in aged neurons.

SASP is the secretion of a variety of molecules such as cytokines, chemokines, proteases, and lipids [16]. One of the major components of SASP, interleukin (IL)-6, not only increases with age but is also released by aged neurons or long-term cultured neurons [3, 25]. *In vitro* experiments demonstrate that functional SASP from long-term cultured neurons results in proliferation of rat astrocytes and senescence of mouse embryonic fibroblasts [16], suggesting the contribution of SASP to paracrine senescence and chronic inflammation [26]. GATA4, a key regulator of SASP, is accumulated in cortical neurons in the old rat brain [8, 16]. It can trigger the expression of a known SASP factor monocyte chemotactic protein 1 (MCP-1). Such paracrine signalling may spread senescence to other cells such as microglia, astrocytes, or endothelial cells. Aged glial cells’ contribution to neuronal senescence is also evident in the aged brain. Aged glial cells contribute and exacerbate neuronal senescence by losing their supportive role for neurons and exerting proinflammatory and neurotoxic effects [27, 28]. For example, some aged astrocytes become reactive astrocytes that are in a more inflammatory state and secrete neurotoxins, while other aged astrocytes and microglia activate complement systems and release proinflammatory cytokines, thus contributing to neuronal senescence and inflammation in the aged brain [29-31]. The presence of these interacting senescent glial cells and neurons may lead to the overall aging of the brain.

Senescent cells go through nuclear morphological changes such as altered shape and size as well as decreased expression of lamin-B1 [32]. Lamin-B1 is an intermediate filament protein that comprises the nuclear lamina beneath the inner nuclear membrane. Loss of lamin-B1 is a senescence-associated biomarker [33], but it is not easy to be detected in the aged brain by immunostaining since mature neurons express a low lamin-B1 level [34]. However, in old rat brains, the altered nuclear envelope still is observed in some cortical neurons [16]. In long-term cultured mouse and rat neurons, abnormal nuclear morphology and decrease of lamin-B1 are observed [16, 35, 36], revealing that nuclear morphological changes occurs in aged neurons.

Lipofuscin is a yellow-brown, auto-fluorescent pigment aggregate consisting of metals (2%), lipids (20-50%), and misfolded proteins (30-70%) [37, 38]. It is mainly accumulated in the lysosomes of post-mitotic cells such as cardiac myocytes and neurons progressively over age [39, 40], hence considered as a consequence of aging. However, this kind of aging-related pigment aggregates is also found in aged glia [7] and other mitotic cells [41]. These aging-related pigment aggregates are also found in the neurons of cerebral cortex and the Purkinje neurons of cerebellum, positively correlating with dominant changes in their neuronal morphologies [16, 39].

DNA damage, dysfunctional mitochondria, SASP, and increased cell-cycle inhibitors are considered to be highly involved in the development of cellular senescence. However, there are also other features not essential for the development of cellular senescence, including nuclear morphological changes, increased level of SA-β-gal, and the accumulation of lipofuscin aggregates, that are often present in neurons from old animals and long-term culture [3, 16, 25, 36].

## 3. Aging changes possibly specific to neuronal senescence

During aging, some neurons manifest senescence changes. Around 30% of the neurons acquire common senescence features in rats during aging [16], though the percentages are lower in humans with 5% of the neurons positive for p16 in normal aging brains and around 20% in AD brains [42]. However, some changes in aging neurons are induced by or associated with stress response, accompanying common senescent traits.

### 3.1. Pigmented accumulations

An obvious phenotypic change during normal aging is the pigmented accumulations, including neuromelanin and Marinesco body (MB) in the norepinephrine neurons of the locus coeruleus and dopaminergic (DA) neurons of substantia nigra pars compacta (SNpc).

Neuromelanin begins to appear in the SN in humans at 3 years of age and accumulates over time [43]. There is no neuromelanin in the rodent SN, but these neurons can accumulate neuromelanin under the challenge of L-DOPA *in vitro* [44], highlighting its role in the age- and stress-dependent responses. The biosynthesis of neuromelanin is driven via iron-dependent oxidation of excess cytosolic dopamine and its precursor L-DOPA. Since neuromelanin is an iron chelating compound, it can reduce iron-mediated oxidative damage [45]. Thus, neuromelanin may play a neuroprotective role against iron overload and progressively accumulate in neurons during aging. However, neuromelanin is reported to cause not only mitochondrial dysfunction via the release of iron to induce oxidative stress [46] but also the decrease in enzymatic activity of 26S proteasome [47]. In addition, excess neuromelanin can activate microglial and further damage DA neurons [48].

MB, a spherical eosinophilic nuclear aggregate [49], is composed of proteins and does not contain carbohydrates, lipids, or nucleic acids. MB is strongly associated with aging, as autopsy studies demonstrate that MB inclusion frequencies increase in elderly individuals [50]. MB accumulation mainly occurs in the ventral tier of the SN. In PD, as the number of DA neurons dies, the frequency of MB thus decreases expectedly [51]. MB may not only be a feature of neuronal aging but also a phenotype of neuronal senescence because the accumulation of MB is often accompanied by a parallel enlargement of the nucleus, a well-established feature of senescence, as well as the accumulation of nucleoplasmic p62 [52], though the exact mechanism of how MB leads to enlargement of nucleus remains to be elucidated, possibly due to defective proteostasis.

The appearance of neuromelanin and MB is the outcome of stress response over aging. While the formation of these pigments may serve as a protective measure, the cells containing the pigments are vulnerable to further insults. That explains why DA neurons decrease in the nonhuman primate midbrain over normal aging [53], neuromelanin-rich DA neurons preferentially degenerate in PD [54], and the norepinephrine neurons in the locus coeruleus degenerate many years before the onset of AD [55]. Thus, neuromelanin and MB, the stress response products, may be regarded as features of neuronal senescence.

### 3.2. Structure alteration

Neurons bear processes (axons and dendrites) for sending and receiving information. Such special architecture renders neurons susceptible to aging- and stress-induced changes. Axons (nerve fibers) function to deliver information in the form of electrical signals from the neuronal cell body to axon terminals, which then synapse with another neuron for signal transmission. Axons are often surrounded by myelin sheaths, a multilayer of proteins and lipids that wraps and insulates axons for proper conduction of electrical signals down the axon. A loss of myelin sheaths with age is associated with the loss of brain volume and cognition decline among the elderly. In the cerebral white matter of healthy populations (aged 18-79 years), myelin water fraction decreases with age, which is considered as a marker for age-related myelin changes [56]. The loss of myelin sheath results in longer nodal and paranodal spaces, which is often seen in optic nerves of aged mice [24]. It is also seen in rhesus monkeys and human during the entire life cycle [57, 58]. Demyelination in aged brain may be due to the senescence of oligodendrocytes, which predisposes the demyelinated axons to further damage [59, 60]. That may explain why there is a decrease in the number of axons [61].

Dendrites are responsible for receiving information through neurotransmitter receptors located in the dendritic spine. In the aged brain of human and nonhuman primates, the length and the number of dendritic branches exhibit an obvious decrease in the prefrontal cortex and hippocampus [62-64]. Importantly, the number of dendritic spines decreases with age in the neocortex, hippocampus, and other subcortical regions in various species [65]. There are three major spine categories: thin, stubby, and mushroom [66]. Mushroom spines usually have large spine heads, while thin spines have small heads and are associated with a high degree of plasticity [67, 68]. During aging, almost all the spine loss is attributed to the loss of the thin spines but not the mushroom or stubby spines in rhesus monkey neurons [69]. In addition, aging leads to bigger diameter and volume of the thin spine heads [69]. These changes in the thin spines may be related to cognition decline. The shape and size of the dendritic spines in neurons are dynamic and activity-dependent. They are regulated by interactions between actin filaments and actin-binding proteins within the spine [70], hinting that aging affects the dynamic of those cytoskeleton, contributing to reduced capacity to generate new spines.

The structural integrity and function of both axons and dendrites largely rely on the transport system which is orchestrated by the dynamic cytoskeleton machinery especially microtubules (MTs) and MT associated proteins. In the mature brain, the MTs are relatively stable, mainly owing to the acetylation of MTs [71]. During aging, microtubules are more likely to experience mechanical stress; hence, the neurons tend to accumulate acetylation as a protective measure. However, hyper-acetylation reduces MT dynamics and impairs axonal transport, as seen in DA neurons of aging mice [72]. Over stabilization of MT results in thicker neurites, fewer branches, and loss of synaptic varicosity [73, 74]. Hence, MT acetylation is regarded as a marker for MT aging [75]. As MT dynamics and transport depend on energy, mitochondrial dysfunction is another major contributor to the axonal changes in normal aging. Stress induces the opening of the mitochondrial permeability transition pore which impairs MT transport [24] and contributes to axonal degeneration [76]. Therefore, many axonal and dendritic changes seen in aging bear senescent features.

### 3.3. Decreased neuronal excitability

Neuronal excitability is the ability to produce a rapid change in membrane voltage by ion fluxes in response to stimulation. Its changes also influence synaptic transmission. Action potential generation is mediated by a large transient sodium influx and a subsequent voltage-dependent potassium efflux. One of the neuronal membrane properties, afterhyperpolarization (AHP), is mediated by the Ca^2+^ dependent, potassium currents. The AHP is increased in aged hippocampal neurons [77]. This larger AHP restricts the membrane potential from reaching the action potential threshold and reduces neuronal firing frequency. Similarly, substantia nigra DA neurons from old mice exhibit larger AHP and slower firing rates and more variable interspike intervals than young mice [78]. Human neurons overexpressing the senescence related factor p16 show a decrease in spontaneous firing [42], revealing a direct correlation between neuronal senescence and reduced excitability. Furthermore, the reduced frequency of action potential in turn affects the efficacy of synaptic transmission.

### 3.4. Declined synaptic transmission

Synaptic transmission is the process by which neurons communicate with each other through synapses. The reductions in synaptic transmission are commonly observed in aged animal brains [79, 80]. Its decrease highly correlates with age-related cognitive deficits. Aside from the structural changes described above, dendritic spines are the sites where synaptic inputs land. The number of spines decreases over age, leading to the gradual loss of afferent inputs [81-83]. As the loss of synapse number in the prefrontal cortex and hippocampus directly contributes to cognitive decline, most published work on synapse loss focuses on the forebrain, showing reduction in synapse numbers in aged neurons [84-86]. However, synapse loss is not limited to these brain regions. In aged rat cerebellum, decreased number of synapses is found in presynaptic termini of Purkinje cells and is compensated by an increase in the size of remaining synaptic components [87]. On the other hand, the vesicle trafficking is key for synaptic transmission. Both vesicle trafficking along axons and synaptic vesicle release depend heavily on ATP produced by mitochondria [88]. In aged neurons, mitochondrial dysfunction reduces ATP generation, which further slows vesicle trafficking and release, decreasing synaptic transmission.

### 3.5. Altered neural plasticity

Synaptic plasticity is the change in the strength of synaptic transmission. Functionally, long-term depression (LTD) and long-term potentiation (LTP) are the classical measures of synaptic plasticity, which is also the basis of learning and memory. The time window between the synaptic inputs and the postsynaptic action potential generation determines whether LTP or LTD is generated. Aged animals show an increased tendency to induce LTD and reverse LTP, leading to the decrease of synaptic transmissions [89]. Morphologically, the postsynaptic dendritic spines often reflect synaptic plasticity. Dendritic spines serve as a site to integrate synaptic inputs and participate in synaptic plasticity. Aged neurons exhibit significant changes in spine size and shape, suggesting that aging also brings the changes to synaptic plasticity. Hence, the age-related changes in the dendritic spines have a major impact on synaptic transmission and plasticity.

## 4. Drivers of neuronal senescence

The cellular and functional changes as well as the biochemical alterations in aged neurons, described above, highlight the unique features of aged neurons as well as their similarity to cellular senescence of mitotic cells. Thus, we propose these aging-induced neuronal changes are potential features of neuronal senescence.

Most common senescent features displayed in mitotic cells are also manifested in aged neurons. Nevertheless, the underlying molecular processes may not always be the same. For example, the accumulation of nucleic acid mutations in mitotic cells is largely due to replication errors, resulting in DNA damage. In the postmitotic neurons, it is likely caused by the mistakes occurring during transcription or DNA repair, which, via yet unknown mechanisms, result in neuronal senescence [90]. The post-mitotic nature, unique structural organization, and special cellular connections of neurons demand a balanced intracellular homeostatic system. Increasing evidence reveals that neurons tend to have misfolded protein accumulation, oxidative products, declined mitochondria function, and abnormal increase of intracellular Ca^2+^ during aging, suggesting that an imbalance of intracellular homeostasis contributes to neuronal senescence.

### 4.1. Redox imbalance

The redox system is balanced by ROS production and antioxidant response. As discussed, the unique structure and functional properties of neurons demand high levels of energy production. Accompanying this is the increased production of ROS which may result in oxidative damage. Carbonyl group modification on proteins is one of the common biomarkers of oxidative stress. Indeed, higher levels of oxidized proteins are present in neurons directly reprogrammed from old fibroblasts compared with those from young fibroblasts [91]. Increased levels of peroxidation of arachidonic acid forms are found in the cytoplasm of neurons in aged human brain [92]. ROS can result in DNA damage, including altered DNA bases, DSBs and SSBs, which is one of the critical mediators of cellular senescence [93]. ROS modifies guanine, resulting in the accumulation of 8-oxoguanine (8-oxoG) lesions in the aged brain [19]. Transcriptome analysis of the human frontal cortex shows that aging leads to the reduction in the expression of a set of genes related to synaptic plasticity. These genes play important roles in synaptic plasticity, vesicular transport, mitochondrial function, induction of stress response, antioxidant, and DNA repair [94]. In accordance, the promoters of downregulated genes are also discovered to have guanine-rich sequences that predispose them to ROS-mediated DNA damage [95].

**Figure 1.**
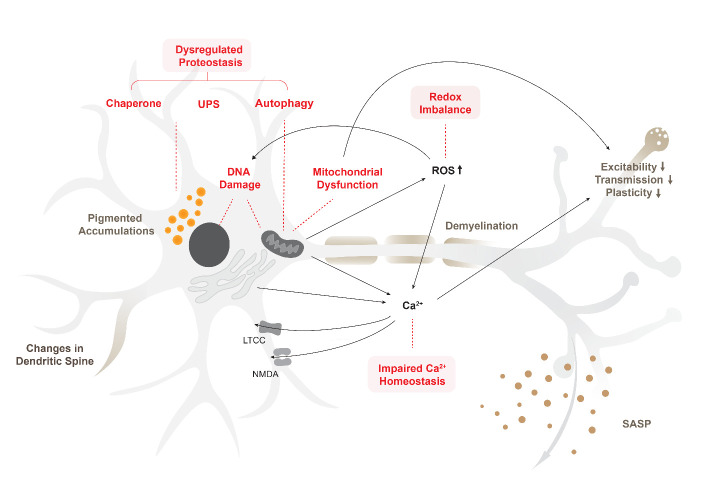
Integrated view of intracellular homeostatic systems leading to neuronal senescence phenotypes. The dysregulated homeostatic systems include impaired proteostasis, which leads to the increase of pigmented aggregations, damaged mitochondria, and release of SASP. Increased ROS as a result of impaired mitophagy, worsened mitochondrial function, and redox imbalance further exacerbate DNA damage and decreased synaptic transmission. Lastly, dysregulated calcium homeostasis from dysfunctional mitochondria, increased LTCC, hypofunctioning NMDA receptors, as well as defective SERCA pumps in the ER contribute to synaptic changes, exacerbating neuronal function. These underlying neuronal senescence mechanisms are reciprocal, creating a vicious loop that ultimately leads to neuronal structural and functional alterations such as changes in the dendritic spine, axonal changes and demyelination, and decreased neuronal excitability.

The main source of ROS is mitochondria. The excess amount of ROS also damages mitochondrial DNA. Since most mitochondrial DNA encodes the proteins of the complex I subunit, the accumulation of mitochondrial DNA damage affects complex I function and impairs the electron transport chain. The consequence is reduced ATP production and increased ROS generation from mitochondria [96], further amplifying oxidative damages in the senescent neurons.

Neurons are equipped with two main antioxidant responses against ROS: Glutathione (γ-glutamyl-cysteinyl-glycine, GSH)-mediated reactions and Nrf2/Keap1 pathway [97]. GSH is an antioxidant in charge of ROS detoxification in neurons [97]. In various animal models, GSH concentration and the ratio of GSH/GSH disulfide decrease with age. The expression of enzymes for GSH biosynthesis decreases in aged rat brains [98]. Reduced neuronal GSH levels increase ROS and lead to Ca^2+^ dysregulation [99, 100]. Nrf2 is a key transcription factor that binds to the antioxidant response element in the promoter of antioxidant-related genes. It regulates the expression of antioxidants and detoxification proteins such as heme oxygenase 1, NAD(P)H dehydrogenase quinone 1, SOD1, GSH peroxidase 1, and catalase [101]. Nrf2 also controls the expression of GSH rate-limiting enzyme glutamate-cysteine ligase catalytic subunit (Gclc). Nrf2 is downregulated in the aging brain [102]. Together, the imbalance of the redox system in neurons over age, compounded by the overproduction of ROS and decline in antioxidant responses, not only leads to progressively accumulated DNA damage but also dysregulates Ca^2+^ homeostasis, resulting in the entry of neurons into the senescent stage (Fig. 1).

### 4.2. Impaired Proteostasis

As post-mitotic cells, neurons robustly manage proteostasis throughout the lifetime of the organism. Three main mechanisms of proteostasis operate in neurons, including re-folding and suppression of protein aggregates by chaperones, degradation by the ubiquitin-proteasome system (UPS), and clearing of abnormal cell contents by autophagy. It is not clear why the efficiency of the proteostasis declines with age, but ribosome pausing at the polybasic regions of many proteostasis-related proteins during translation elongation may be one of the reasons [103]. Molecular chaperones are a family of proteins that facilitate protein folding, assembly, and disassembly; hence they play a central role in the proteostasis system. A comprehensive analysis shows that there are 332 chaperone-related genes in human among which 101 decrease in the brains of older individuals [104], hinting that the protein folding ability declines during neuronal aging.

Autophagy is a self-clearance process that delivers substrates to lysosomes for degradation. It degrades long-lived/insoluble proteins and organelles, accounting for 10~20% of proteolysis. Autophagy-mediated recycling of essential components also serves to supply neurons’ high energy demands [105]. Analysis of autophagy-related genes in young versus aged human brain tissues revealed a decline in the expression of autophagy-related protein 5 (ATG5) and ATG7 [106], key proteins in autophagosome formation. The age-related decline in autophagosome formation may be associated with the abnormal autophagic vesicles with a multilamellar (onion skin-like) structure in the dorsal root ganglion neurons of old mice [107]. The excess membrane accumulation within the autophagic vacuole prevents the recruitment of autophagosome structural protein, LC3B, thus stalling the formation of autophagosomes. As in mitotic cells, autophagy actively degrades GATA4 and suppresses cellular senescence; hence, autophagic dysfunction facilitates the GATA4-mediated SASP [108]. In addition, autophagosomes and autophagic cargo p62 accumulate in rat senescent neurons after long-term culture, consistent with the observation in old rat brains [16]. Inhibition of autophagy leads to more neurons with increased SA-β-gal activity [16], revealing that dysfunctional autophagy contributes to neuronal senescence. On the contrary, enhancing autophagy by mTOR inhibitor reduces protein aggregates and senescence markers in rat neurons after long-term culture [36]. These evidences support a clear correlation between autophagy failure and senescence conversion in neurons.

Autophagy-mediated degradation of impaired mitochondria, or mitophagy, is necessary for maintaining normal cell metabolism. The damaged mitochondria are labeled by Parkin, an E3 ubiquitin ligase, which will undergo mitophagic degradation. Neuronal activities, including neurotransmission along axons and synaptic vesicle release, rely heavily on ATP produced by mitochondria [88]. Dysfunctional mitophagy may impair signal transduction along the axons and/or synaptic vesicle recycling at the presynaptic end seen in senescent neurons, impacting neural transmission and hence cognitive function [23] (Fig. 1).

The majority (80~90%) of proteolysis is carried out by the UPS that removes ubiquitylated short-lived/soluble proteins [109, 110]. Ubiquitinated proteins are degraded by the proteosome. In rats, the percentage of proteosome in total brain protein is lower in aged brains than young brains (19% less in cerebrum, 31% less in cerebellum, and 37% less in hippocampus) [111]. Not only the amount but also the activity of the proteosome is reduced in aged mouse and rat brain, such as the cortex, cerebellum, SN, striatum, and globus pallidus in aged rats and mice [112]. The dysfunctional UPS/proteosome activity may account for the pigmented aggregates in neurons, including MB, lipofuscin, and neuromelanin complex [52, 53, 113, 114]. MB is immunoreactive to ubiquitin and contains some other proteins of the UPS, suggesting that its formation is involved in the progressive functional decline of nuclear UPS with advanced age [113]. Lipofuscin is a highly oxidized aggregate. Due to its covalently cross-linked nature, lipofuscin is difficult to be cleaned by the UPS but is up-taken by autophagosomes and accumulated in lysosomes [115]. Neuromelanin-iron complexes include metals, lipids, and proteins, and they accumulate inside neuronal organelles and lysosome, interfering with their function. Analysis of neuromelanin complex and lipofuscin isolated from human neurons reveals membrane, cytoskeleton, mitochondrial, and vesicle protein contents [43, 116], revealing that lysosomal cargo overload occurs in the lysosome of senescent neurons. Thus, imbalanced proteostasis, especially impaired autophagy and UPS, is associated with the accumulation of undegradable aggregates in the lysosome in senescent neurons (Fig. 1).

### 4.3. Dysregulated Ca^2+^ homeostasis

Ca^2+^ homeostasis is essential for cellular functions, especially neuronal excitation. The concentration gradient of Ca^2+^ is very steep (10,000-fold) between extracellular (1.5 to 2 mM) and cytoplasm (50 to 100 nM), therefore, adequate intracellular Ca^2+^ concentrations must be maintained for appropriate neuronal function [117]. In neurons, Ca^2+^ contributes to synaptic activity as well as the transmission of the depolarizing signal [118]. The recovery of intracellular Ca^2+^ following such activation-evoked Ca^2+^ signaling is an energy-consuming process involving specific Ca^2+^ pumps or transporters. To maintain intracellular Ca^2+^ homeostasis, Ca^2+^ pumps are utilized to transport cytosolic Ca^2+^ either to the extracellular space or into the endoplasmic reticulum (ER). The ER extends from the nucleus to the soma, dendrites, as well as the axon, and acts as a storage pool of Ca^2+^, which dynamically controls the accumulation and release of Ca^2+^ in response to stimulation. Through the sarco/endoplasmic reticulum Ca^2+^-ATPase (SERCA) pumps, ER can restore the resting cytoplasmic Ca^2+^ concentration after neuronal activation [119]. However, the activity of the SERCA pumps is reduced in older neurons relative to young neurons [120]. Consequently, it takes a longer time to remove excess Ca^2+^ in the cytoplasm in the older neurons than young neurons, resulting in elevated Ca^2+^ currents [121]. Excess intracellular Ca^2+^ can inhibit sodium flow through voltage-gated sodium channels, leading to decreased depolarization and further impact action potential formation.

A postsynaptic increase in intracellular Ca^2+^ is required to trigger the initiation of signaling cascades for LTP or LTD [122]. The spatiotemporal nature and amplitude of Ca^2+^ signals determine the fate of synaptic plasticity (LTP or LTD). Weak presynaptic stimulation leads to a modest Ca^2+^ influx through NMDA receptors in postsynaptic spines, which triggers LTD. Strong presynaptic stimulation leads to LTP via high postsynaptic Ca^2+^ influx. The influx of Ca^2+^ in postsynaptic sites is mediated by the NMDA receptors. In old hippocampal neurons, redox-mediated hypofunction of NMDA receptor decreases Ca^2+^ influx [123], favoring LTD over LTP generation. In addition, the cytoplasmic Ca2+ level is also regulated by the L-type Ca^2+^ channel (LTCCs). In an aged hippocampus, CA1 pyramidal neurons exhibit an increased density of LTCCs. Increased number of LTCCs bring an extra amount of Ca^2+^ in neurons [124], altering neuronal excitability and synaptic plasticity. As discussed earlier, AHP is Ca^2+^-dependent. The increased number of LTCCs in senescent neurons [125, 126] elevates Ca^2+^ influx and potentiates the amplitude and duration of the AHP [127], reducing excitability and impairing neural transmission/plasticity (Fig. 1).

The cytoplasmic Ca^2+^ level is also regulated by mitochondria via the Ca^2+^ uniporter. Mitochondria have a huge transport capacity (maximum transport capacity to the mM range) with an optimal affinity at the µM range at the peak of Ca^2+^ responses [128]. Neuronal stimulation enhances mitochondrial Ca^2+^ uptake from the cytosol for depolarization. *In vitro* studies demonstrated that mitochondrial repolarization in response to stimulation is delayed in old neurons due to limited mitochondrial Ca^2+^ uptake capacity [23]. Dysfunctional mitochondria contribute to the poor regulation of intracellular Ca^2+^ in neurons [129], dysregulating neuronal function (Fig. 1).

## 5. Implications of neuronal senescence

### 5.1. Intracellular homeostatic systems

Being postmitotic, neurons must maintain the integrity of the complex structure and at the same time operate the precise communication with their partners throughout the life of an organism without the ability to get rid of accumulated junks through cell division. Hence, neurons must endure enormous stress to maintain their structural integrity and physiological operation. We propose neuronal senescence as the outcome of adaptation of the intracellular homeostatic systems to endure stress, including proteostasis, redox balance, and Ca^2+^ homeostasis. Both the structural maintenance and physiological operation of a neuron demand high energy production, which is accompanied by over generation of ROS. While the neurons are equipped with the antioxidant machinery, over time (during aging), the redox balance is tippled toward oxidation. The imbalanced redox not only impairs mitochondrial integrity and function, resulting in further ROS production, but also oxidizes intracellular molecules like neurotransmitters and dysregulates proteostasis as well as causes nuclear and mitochondrial DNA damage. That explains why the neuronal pigmented accumulations contain oxidative protein deposits. Furthermore, the imbalanced redox environment favors the release of Ca^2+^ from the ER and mitochondria. Together with the increased entry of Ca^2+^ from the extracellular space via LTCC and NMDA receptors, it raises the cytoplasmic level of Ca^2+^. The dysregulated Ca^2+^ homeostasis alters the membrane properties and synaptic vesicle release, impairing neuronal excitability, neuronal transmission and neural plasticity that are seen in senescent neurons. The redox balance and Ca^2+^ homeostasis are also intertwined with other intracellular homeostatic systems, especially the proteostasis system. As part of the proteostasis system, impaired UPS, especially lysosomal overload or lysosomal function decline, is associated with the accumulation of protein aggregates and dysfunctional mitochondria. In addition, a functional decline of autophagy exacerbates redox imbalance and enhances SASP to spread senescence. These aging-induced functional declines of intracellular homeostatic systems contribute to the accumulation of misfolded proteins, oxidative products, and DNA damage, eventually leading to neuronal senescence.

### 5.2. Models for human neuronal senescence

A good model for human aging-induced neuronal senescence should have measurable senescence phenotypes and ideally identifiable conserved biochemical mechanisms. Animal models are invaluable to identify the role of individual genes and epigenetic phenomena in aging. Widely studied model organisms in aging includes yeast, C. elegans, Drosophila, rodents, and non-human primates. The advantage of these models is their genetic relevance to humans, short generation time, and ease of manipulation, especially genetic engineering. Interesting, long-term culture of animal neuronal cells is a quite well-described model of neuronal senescence since they can display senescence-like phenotypes [25, 130], illustrating that time is one of the critical factors for neuronal senescence. However, it is unclear whether mechanisms of aging studied in short-lived organisms is transferable to long-lived humans [131, 132].

Access to viable human brain samples is limited. Single cell transcriptomic profiling of postmortem brain samples of different ages gives a glimpse of the molecular phenotypes and potential pathways involved in neuronal aging [133-135]. A great interest in neuroscience is the use of reprogrammed human cells as a living model for studying neuronal senescence and degeneration. Direct conversion of fibroblasts to induced neurons retains age-related epigenetic marks, permitting analysis of age effects on neuronal activity and its relationship with neurodegeneration [136, 137]. However, direct conversion is low throughput. Directed differentiation of neurons from induced pluripotent stem cells (iPSCs) may overcome this issue. However, the reprogramming process of iPSC generation erases the age-related epigenetic marks; hence, the neurons differentiated from iPSCs display embryonic phenotypes. One way to induce senescence is to introduce progerin [138] or knockout SATB1 [139]. The DA neurons generated from these progerin overexpressing or SATB1 knockout iPSCs indeed exhibit senescent phenotypes, including enhanced nuclear folding and blebbing, neuromelanin accumulation, increased mitochondrial ROS, impaired lysosomal and mitochondrial function, DNA damage accumulation, and dendrite degeneration. However, the caveat is the difficulty in separating the effect of the transgene on senescence from degeneration. An alternative method is to employ non-genetic means. Fathi et al. screened small molecules that regulate the pathways described above and developed a cocktail that induces senescent phenotypes in fibroblasts and different types of neurons derived from human embryonic stem cells (ESCs) and iPSCs. Importantly, when induced with the “senescence cocktail”, the amyotrophic lateral sclerosis iPSC-derived motor neurons display degenerative phenotypes including protein aggregation and neurite degeneration within a week [140], significantly facilitating the phenotypic presentation. Similarly, Hergenreder et al. used a high-content imaging assay to identify compound cocktail capable of accelerating the maturation of cortical neurons derived from human iPSCs [141]. This cocktail can promote the maturation of several human iPSC-derived cell types, including cortical neurons, spinal motoneurons, melanocytes, and pancreatic beta cells. These strategies summarized here will likely enhance disease modeling and drug testing using reprogrammed neurons.

### 5.3. Contribution of neuronal senescence to neurodegeneration

Aging is the major risk factor for the development of neurodegenerative diseases such as PD, HD, and AD. Aging undermines brain’s capacity to cope with glucose availability, mitochondrial dysfunction, vascular dropout, and inflammatory stress, which are underlying factors for neurodegeneration [142], placing neurons under the stress conditions, very much like the dysfunctional homeostasis systems described above for senescent neurons. Huntingtin, a scaffold protein involved in selective autophagy, regulates the interaction of cargo receptor p62 with LC3B and lysine-63-linked ubiquitin-modified substrates [143]. Mutations in huntingtin reduce the motility of autophagosomes, contributing to the pathogenesis of HD [105]. Abnormal PINK1 destabilizes Parkin and damages mitophagy by reducing the activity of ubiquitin ligase, contributing to PD [144]. In AD neurons, Aβ oligomers abnormally interact with dynein, thus restricting autophagosomes to distal axons and impairing their degradation in the soma [145]. The imbalanced homeostasis systems in neurons, triggered by genetic and/or environmental factors, result in the initiation and/or progression of neurodegeneration. Hence, neurodegeneration may be viewed as the amplification of the neuronal senescence phenotypes, including misfolded protein inclusions, mitochondrial dysfunction, and altered synaptic transmission [146]. On the other hand, in neurodegenerative diseases, neurons undergo neurodegeneration, which is accompanied by cell death. Senescence is a protective mechanism for a cell to prevent further spread of damage by arresting the cell cycle without cell death. The percentage of senescent neurons in AD brains is higher than in normal aging brains, hinting that neuronal senescence may serve similar roles as mitotic senescence to avoid neurons’ sudden death. The etiology of neurodegenerative diseases remains poorly understood, further investigating why patients have more senescent neurons will help us find out how these diseases develop. It may also guide us to develop approaches to decelerate the so far inevitable aging process to delay or slow the development of neurodegenerative diseases.

## 6. Conclusions and Perspectives

Aging, neuronal senescence, and degeneration are a continuum. Overlapping phenotypes and mechanisms involved in neuronal aging and senescence make it difficult to delineate and clearly define that these phenotypes/mechanisms are specific to aging or senescence. Nevertheless, accumulating studies show that some aging changes are possibly specific to neuronal senescence as they are closely associated with stress response and accompanied by common senescence markers, and they ultimately result in degeneration. Thus, we propose that age-induced changes such as declines in proteostasis, redox imbalance, and altered calcium homeostasis contribute to and exacerbate the stress that pushes neurons into obtaining senescence phenotypes. They include the accumulation of stress-related products such as DNA damage, misfolded proteins, and pigmented macromolecules (lipofuscin, neuromelanin, and MB) in neurons. Some of these products are well-known markers for cell senescence, but neuronal senescence cannot be determined by a single phenotype. It is important to identify more specific markers for senescent neurons, particularly in the changes of neuronal structure and function mentioned above. Neuronal senescence may be viewed as a protective measure to prevent the spread of senescence to other cells. Hence, understanding how a neuron acquires cellular senescence and ultimately degenerate is beneficial for us to prevent or slow the process of neuronal senescence and degeneration.
